# Paeoniflorin inhibits excitatory amino acid agonist-and high-dose morphine-induced nociceptive behavior in mice via modulation of N-methyl-D-aspartate receptors

**DOI:** 10.1186/s12906-016-1230-x

**Published:** 2016-07-26

**Authors:** Yuh-Fung Chen, Ming-Ming Lee, Hsun-Lang Fang, Jhao-Guei Yang, Yu-Chien Chen, Huei-Yann Tsai

**Affiliations:** 1 Department of Pharmacology, China Medical University, No 91, Hsueh-Shih Road, Taichung, 40402 Taiwan; 2 Department of Pharmacy, China Medical University Hospital, No 2, Yu-Der Road, Taichung, 40431 Taiwan; 3 Department of Health and Nutrition Biotechnology, Asia University, No 500 Lioufeng Road, Wufeng District, Taichung, 41354 Taiwan; 4 Laboratory of Computational and System Biology, China Medical University, Taichung, No 91, Hsueh-Shih Road, Taichung, 40402 Taiwan; 5 Department of Biomedical Informatics, Asia University, No 500 Lioufeng Road, Wufeng District, Taichung, 41354 Taiwan

**Keywords:** Paeoniflorin, Excitatory amino acid agonists, High-dose morphine, Nociceptive behavior, Antisense oligodeoxynucleotides, NMDA receptor, NR2B

## Abstract

**Background:**

Pain, the most common reasons for physician consultation, is a major symptom in many medical conditions that can significantly interfere with a person’s life quality and general functioning. Almost all painkillers have its untoward effects. Therefore, seeking for a safe medication for pain relieve is notable nowadays. *Paeonia lactiflora* is a well-known traditional Chinese medicine. Paeoniflorin is an active component found in *Paeonia lactiflora*, which has been reported to inhibit formalin-induced nociceptive behavior in mice. Aims of this present study were to investigate effects of paeoniflorin on excitatory amino acid agonist- or high-dose morphine-induced nociceptive behaviors in mice.

**Results:**

Paeoniflorin (100, 200, 500 nmol, i.c.v.) alone and combined with glutamatergic antagonists (MK-801 14.8 pmol, or NBQX 5 nmol, i.t.) inhibited nociception. Those agents also inhibited the clonic seizure-like excitation induced by high-dose morphine (250 nmol, i.t) in mice. Antisense oligodeoxynucleotides of NMDA receptor subunits NR1, NR2A, NR2B significantly enhanced the inhibition of paeoniflorin on excitatory amino acid-and high-dose morphine-induced nociception. Docking energy data revealed that paeoniflorin had stronger binding activity in NR2A and NR2B than NR2C of NMDA receptors.

**Conclusions:**

Results of this study indicate that paeoniflorin-induced inhibition of excitatory amino acid agonist- and high-dose morphine-induced nociceptive behaviors might be due to modulation of NMDA receptors, specifically the NR2B subunit.

## Background

Glutamate is an excitatory amino acid (EAA) and a crucial neurotransmitter involved in nociceptive signaling and pain modulation in the central nervous system [1–5]. Glutamate and glutamatergic receptors locate in the central and peripheral nervous systems [6, 7]. The excitation of glutamate is partially mediated through the ionotropic glutamate receptors (iGluRs) including *N*-methyl-D-aspartate (NMDA), α-amino-3-hydroxy-5-methylisoxazole-4- propionic acid (AMPA), and kainate receptors [8, 9]. NMDA receptors, among the iGluRs, have received the most attention for they mediate central and peripheral sensitization during pain states [10, 11]. Results obtained from animal behavioral studies suggest that NMDA receptor antagonists have antinociception effects [12–14]. Pretreatment with NMDA receptor antagonist effectively attenuates formalin-induced nociception in rats [15]. Besides, experiments show that high doses of morphine produce hyperalgesia, allodynia and myoclonic seizures in mice and rats [16–18]. Morphine-induced hyperalgesia is a state of nociceptive sensitization caused by exposure to morphine [19]. It has been reported that such effects are mediated by NMDA receptor (NMDAR) and can be reversed by an NMDA receptor antagonist but not by naltrexone [20].

NMDAR is composed of the NR1, NR2 (A, B, C, and D) and NR3 (A and B) subunits. Several reports are indicating that the NR2B subunit is involved in mediating pain [21–23] and learning. It is well-established that the NMDA receptor NR2B subunit is an important contributor to pain mechanisms. Identifying selective NR2B antagonistic drugs is a priority in pain management [24].

**Fig. 1:**
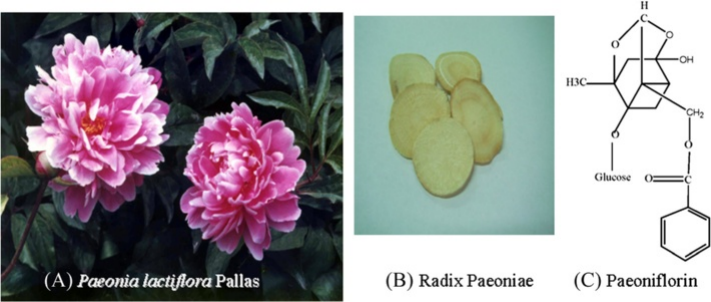
Plant (**a**) medicinal part (**b**) and the active component (**c**) of *Paeonia lactiflora* Pallas

*Paeonia lactiflora* Pallas is an ornamental and a medicinal herb (Fig. 1a). The medicinal part is the dry root (Fig. 1b). The chemical structure of paeoniflorin, 1 of its active components, is shown in Fig 1c. Paeoniflorin (PF) is a water-soluble monoterpene glycoside that has anti-oxidant, anti-inflammatory [25–29] and analgesic effects [30, 31]. An earlier report indicated that PF reduced formalin- and acetic acid-induced nociception in mice [32]. Activation of NMDA receptors during formalin-induced nociception has been reported [33]. Antisense oligonucleotide to NMDA receptor subunits attenuate formalin-induced nociception and knock down of spinal NMDA receptors reduces NMDA and formalin evoked behaviors in rat [34, 35]. In our preliminary study, PF significantly potentiated the antinociceptive effect of MK-801, an NMDA receptor antagonist, in formalin-induced nociceptive behavior in mice. It indicates that the antinociception effect of PF may involve an interaction with glutamatergic receptors, specifically the NMDA receptor. In this study, actions of PF on NMDA receptor function were investigated using the formalin test, and excitatory amino acid (EAA) agonist- and high-dose morphine- induced biting and scratching behavior in mice. The antisense oligodeoxynucleotides (ODNs) of the NMDA receptor subunits were used to study the potential mechanisms of action of PF on EAA- and high-dose morphine- induced nociceptive behavior in mice.

## Methods

### Chemicals

Paeoniflorin (PF) was purchased from Nacalai tesque (Kyoto, Japan). L-Glutamic acid hydrochloride (glutamate), *N*-methyl-D-aspartic acid (NMDA), (±)-α-amino-3-hydroxy-5-methylisoxazole-4-proprionic acid hydrobromide (AMPA), (5R,10S)-(+)-5-methyl-10,11-dihydro-5H-dibenzo [*a*,*d*] cyclohepten-5,10-imine hydrogen maleate (MK-801), 2,3-Dioxo-6-nitro-1,2,3,4-tetrahydrobenzo [*f*] quinoxaline-7-sulfonamide disodium (NBQX) were purchased from Research Biochemical Incorporated (Natick, MA, USA). Anti-rabbit HRP-IgG was purchased from Abcam (Cambridge, UK). Morphine hydrochloride was purchased from Food and Drug Administration, Ministry of Health and Welfare (Taipei, Taiwan). Formalin was purchased from Merck (Darmstadt, Germany). Antisense ODN subunits: NR1, NR2A, NR2B, NR2C, and β-actin were purchased from Sigma-Aldrich (St. Louis, MO, USA). Zoletil® was purchased from Virbac Laboratories (Carros, France). The anti-NR2B antibody was purchased from R & D Systems (Minneapolis, MN, USA).

### Animals and treatment

Male ICR mice (18–25 g) were purchased from the National Laboratory Animal Center, Taipei, Taiwan. Mice were housed 5 per cage at a constant temperature (22 ± 1 °C) and relative humidity (60 %) under a regular light–dark schedule (light 7:00 AM to 7:00 PM) and with free access to food and water. Each animal was used only once. The experimental protocol was approved by Animal Care and Use Committee, China Medical University (permit number 102–224) and conducted in agreement with the Ethical guidelines for investigations of experimental pain in conscious animals [36]. All drugs for intrathecal (i.t.) and intracerebroventricular (i.c.v.) administration were dissolved and diluted to appropriate concentrations in artificial CSF (ACSF), except that formalin solution was diluted with normal saline. Drug solutions were freshly prepared before the experiment and given in a volume of 5 μl in ACSF.

### Drug administration schedule

**Fig. 2:**
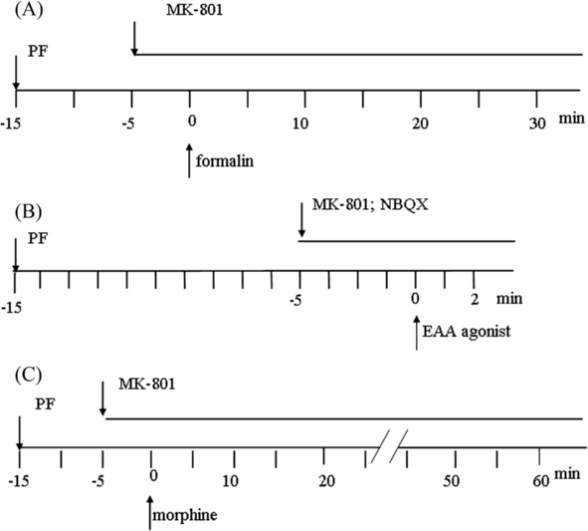
Schedule of drug treatment and experimental processes. (**a**) PF (100, 200, 500 nmol, i.c.v.) was administered 15 min before intraplantar injection of 20 μl 1% formalin. MK-801 (14.8 pmol, i.t.) was injected 5 min before intraplantar formalin injection. (**b**) PF (100, 200, 500 nmol, i.c.v.) was administered 150min before intrarthecal injection of EAA agonists (in 5 μl ACSF). MK-801 (14.8 pmol, i.t.) and NBQX (5 nmol) were injected 5 min before intrathecal injection of EAA agonists. The time spnet on biting or scretching induced by EAA agonists was observed and recorded during the first 2 min. (**c**) PF (100, 200, 500 nmol, i.c.v.) were administered 15 min and 5 min intrathecal injection of morphine, respectively. Mice were intrathecally administered morphine (250 nmol). The onset and the total number of clonic seizure-like excitatory behavior were observed and recorded for 60 min

Glutamate 500 nmol, NMDA 122 pmol, AMPA 12.5 pmol [8, 37, 38], MK-801 14.8 pmol, NBQX 5 nmol, morphine 250 nmol [39], and PF 100, 200, 500 nmol [32] were used. The drug administration schedule is shown in Fig. 2.

### Effects of PF on formalin-induced licking and biting behavior

Mice were allowed to acclimatize for 30 min before drug injection. Twenty μl of formalin (1 %) was intraplantar injected into the dorsal surface of the hind paw of the mouse using a 31 gauge needle. Immediately after a formalin injection, animals were individually placed in the observation chamber, and a mirror was arranged at a 45° angle under the cage to allow clear observation of the paws of the animals. PF (100, 200, 500 nmol, i.c.v.) was administered 15 min before intraplantar injection of 20 μl 1 % formalin. MK-801 (14.8 pmol, i.t.) was injected 5 min before intraplantar formalin injection (Fig. 2a). The total time (seconds) spent on licking and biting of the injected paw during periods of 0–5 min (early phase), and 10–30 min (late phase) were measured as indicators of nociceptive behavior [40, 41].

### Effects of PF on EAA agonists-induced biting and scratching behavior

Intrathecal injections of EAA agonists, such as glutamate (500 nmol), NMDA (122 pmol) and AMPA (12.5 pmol), result in behavior characterized by caudally directed biting and scratching in mice [2, 37, 38, 42, 43]. PF (100, 200, 500 nmol, i.c.v.) was administered 15 min before intrathecal injection of EAA agonists (in 5 μl ACSF). MK-801 (14.8 pmol, i.t.) and NBQX (5 nmol) were injected 5 min before intrathecal injection of EAA agonists. After intrathecal injection of EAA agonists, each mouse was then placed in the observation chamber. The time spent on biting or scratching induced by EAA agonists was observed and recorded during the first 2 min (Fig. 2b).

### Effects of PF on the high dose morphine-induced clonic seizure-like excitatory behavior in mice

PF (100, 200, 500 nmol, i.c.v.) and MK-801 (14.8 pmol, i.t.) were administered 15 min and 5 min before intrathecal injection of morphine, respectively. Mice were intrathecally administered morphine (250 nmol) and were immediately placed in an observation chamber. The onset and the total number of clonic seizure-like excitatory behavior were observed and recorded for 60 min [39]. The of drug treatment schedule is shown in Fig. 2c.

### Effects of PF and antisense ODN subunits on the high dose morphine-induced clonic seizure-like excitation behavior

Mice were administered various antisense ODNs: NR1, NR2A, NR2B, and NR2C (15nM dissolved in ACSF, i.c.v.) 24 h before injection of morphine (250 nmol, i.t.). Different concentrations of PF (100, 200, 500 nmol, i.c.v.) were administered 15 min before morphine administration. The onset and the total number of clonic seizure-like excitatory responses in mice were observed and recorded for 60 min.

### Effects of PF and antisense ODN subunits on NMDA-induced biting and scratching behavior

Mice were administered various antisense ODNs: NR1, NR2A, NR2B, and NR2C [44] (15nM dissolved in 5 μl ACSF, i.c.v.) once a day for 1, 3 and 7 days. NMDA (122 pmol, i.t.) was administered. Different doses of PF (100, 200, 500 nmol) were used to investigate the effect of antisense ODN subunits on NMDA-induced biting and scratching behavior. The onset and the total number of biting and scratching behavior in mice were observed and recorded for 2 min.

### Effects of PF and antisense ODN of NR2B subunit on NMDA receptor in brain cortex by immunohistochemical detection

Mice were deeply anesthetized by an intraperitoneal injection 50 mg/kg of zoletil® and sacrificed. Brain cortex was quickly removed and soaked in 4 % paraformaldehyde to dehydrate and fix for overnight to form a paraffin-embed tissue. Tissue was sliced into 5 μm thickness with a microtome. Brain slices were incubated with the anti-NMDA receptor subunit 2B (NR2B) antibody (R&D Systems, Minneapolis, MN, USA) overnight and immunohistochemical labeled using a NovoLink Polymer Detection System Kit (Leica Microsystems Inc., Newcastle Upon Tyne, UK). The positive NR2B staining cells within the cortex were detected and photographed using an inverted microscope as previously described [45].

### Effects of PF and antisense of ODN NR2B on protein levels of NMDA Receptor in mouse brain by western blotting

Mice were anesthetized and sacrificed. Brain tissue of mouse was quickly removed on the ice. A 10 % homogenate was prepared in lysis buffer, centrifuged at 13,000 (rpm) for 15 min at 4 °C. Total protein was prepared with RIPA protein lysis buffer, and the concentration of protein was determined by the Bradford method using Bio-Rad protein assay dye reagent (Amresco, OH, USA). SDS-PAGE separated the cell lysates containing 30 μg of protein and transferred to a polyvinylidene fluoride (PVDF) membrane (Millipore, MA, USA). Five percent non-fat milk in PBST buffer was used to block non-specific binding sites. The PVDF membranes were incubated overnight at 4 °C with specific primary antibodies for NR2B (R&D Systems, Minneapolis, MN, USA) and β-actin (Sigma-Aldrich, MO, USA). The membranes were then washed with PBST buffer and incubated with horseradish peroxidase-conjugated secondary antibodies (Santa Cruz Biotechnology, CA, USA). Immunoreactive proteins were detected using a Western Blotting Chemiluminescence Reagent Plus kit (Millipore, MA, USA) and developed on Kodak Bio-MAX light film (Eastman Kodak, Rochester, NY, USA) as previously described [46].

### Effects of PF docked in the active sites of NMDA receptor subtypes

Each simulation and calculation was launched under Discovery Studio (Discovery Studio Modeling 2.0, Accelrys, San Diego, CA, USA). The receptor structure of NMDA including NR1, NR2A, and NR2B were obtained from protein data bank (PDB ID: 1Y1M, 2A5S, and 1FTK, respectively). Waters and ligands were removed from the crystal structures before the minimization of whole crystal structure and the docking of the ligand to the receptors. Paeoniflorin was prepared (ChemOffice 2006, Cambridge Scientific Computing, Cambridge, Massachusetts, USA), including sketch and minimization (MM2 force field) before docking procedure. After the comparable performance, the protocol of Dock Ligands (Ligandfit) was used to predict the binding mode of this compound, and the scoring functions of the docking results were calculated automatically.

Binding free energy between ligand and receptor was calculated under the Chemistry at Harvard Macromolecular mechanics (CHARMm) force field according to the following equation:

$$ {\mathrm{E}}_{\mathrm{interaction}} = {\mathrm{E}}_{\mathrm{complex}}\hbox{--}\ \left({\mathrm{E}}_{\mathrm{ligand}} + {\mathrm{E}}_{\mathrm{receptor}}\right) $$

Where E is energy. The lower binding free energy demonstrated, the better stability.

### Statistical analysis

Results are expressed as mean ± standard error. Data were analyzed by one-way ANOVA followed by Dunnett’s test. *P* < 0.05 was considered significant.

## Results

### Effects of PF and MK-801 on formalin-induced licking and biting behavior

**Fig. 3:**
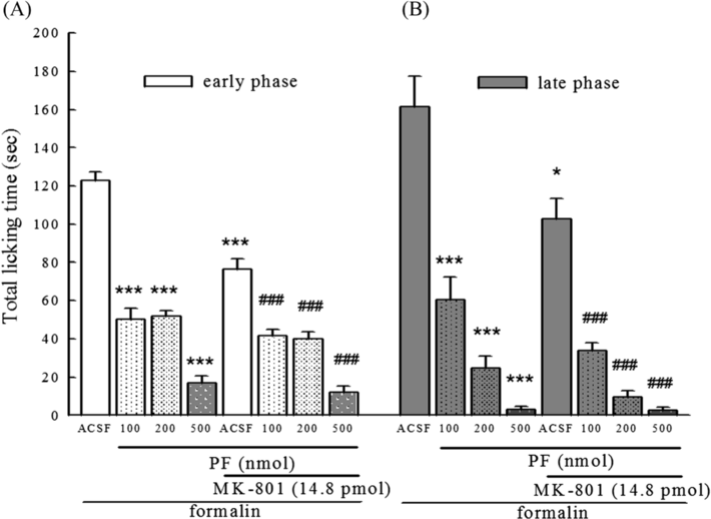
Effects of PF and MK-801 on formalin-induced licking and biting behavior in mice. **a**: represents early phase (0–5 min) and **b** represents late phase (10–30 min) of formalin-induced nociception. PF (100, 200, 500 nmol, i.c.v.) was administered 15 min before intraplantar injection of 20 μl 1 % formalin. MK-801 (pmol, i.t.) was injected 5 min before intraplantar formalin injection. The control group received ACSF. Total time of each mouse spent on licking and biting of the injected paw was recorded. Data represents mean ± S.E. (*n* = 12). ^***^ *p* < 0.001 compared with the control group; ^###^ *p* < 0.001 compared with the MK-801-treated group, respectively

Figure 3 shows that different concentrations of PF (100, 200, 500 nmol, i.c.v.) caused significant inhibition in both early (0–5 min) and late (10–30 min) phases of formalin-induced licking and biting behavior of mice. Co-administration of PF significantly enhanced inhibitory effects of MK-801 on formalin-induced licking behavior at the late phase (*p* <0.001).

### Effects of PF Combined with MK-801 and NBQX on EAA agonists-induced biting and scratching behavior

**Fig. 4:**
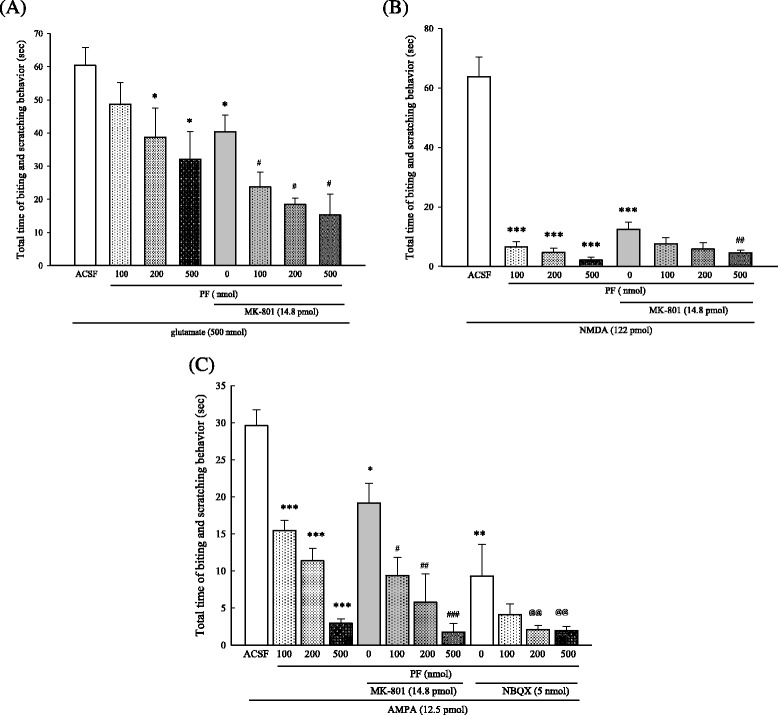
Effects of PF, MK-801, and NBQX on EAA agonist-induced biting and scratching behavior in mice. **a**: treatment with PF + MK-801 on glutamate-induced behavior; **b**: treatment with PF + MK-801 on NMDA-induced behavior; **c**: treatment with PF + MK-801 or PF + NBQX on AMPA-induced behavior. PF (100, 200, 500 nmol, i.c.v.) was administered 15 min before excitatory amino acid injection. MK-801 (MK 14.8 pmol, i.t.) and NBQX (5 nmol, i.t.) were administered 5 min before excitatory amino acid injection. The control group received ACSF. The time spent on biting or scratching induced by excitatory amino acids during the first 2 min was recorded. Data represent mean ± S.E. (n = 12). ^*^ *p* < 0.05, ^***^ *p* < 0.001 compared with the control group; ^#^ *p* < 0.05, ^##^ *p* < 0.01, ^###^ *p* < 0.001 compared with the MK-treated group, respectively

Five μl of EAA agonists, such as glutamine (500 nmol), NMDA (122 pmol) and AMPA (12.5 pmol), were intrathecally administered to evoked biting and scratching behavior, respectively. It showed that co-administration of PF (100, 200, 500 nmol, i.c.v.) produced a dose-dependent inhibition of glutamate-, NMDA-, AMPA-induced biting and scratching behavior (Fig. 4a, b, and c). In the NMDA-induced nociceptive behavior, PF showed significant dose-related attenuation (*p* < 0.001). Co-administration of 500 nmol of PF and MK-801 (14.8 pmol) displayed a 71 % augment of the inhibition of MK-801 on NMDA-induced nociceptive behavior (Fig. 4b). In addition, co-administration of PF (200, 500 nmol) and MK-801 demonstrated a 68 % and 90 % augment of the inhibition of MK-801 on AMPA-induced nociceptive behavior, respectively (Fig. 4c, *p* < 0.01 and *p* < 0.001). Co-administration of PF (200, 500 nmol) and NBQX (5 nmol) demonstrated an 80 % and 80.5 % augment of the inhibition of NBQX on AMPA-induced nociceptive behavior, respectively (Fig. 4c, *p* < 0.01).

### Effects of PF with MK-801 on high dose morphine-induced clonic seizure-like excitatory behavior

**Fig. 5:**
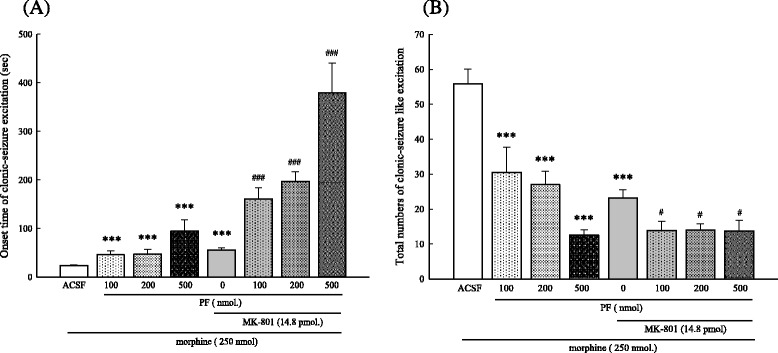
Effects of PF and MK-801 on high dose morphine-induced clonic seizure- like excitation. PF (100, 200, or 500 nmol, i.c.v.) was administered 15 min before injection of morphine (250 nmol, i.t.). MK-801 (14.8 pmol, i.t.) was injected 5 min before excitatory amino acid injection. Control mice received ACSF. The onset time **a** and total numbers of clonic seizure-like excitation episodes **b** induced by morphine injection was recorded for 60 min. Data represent mean ± S.E. (*n* = 12). ^***^ *p* < 0.001 compared with the control group; ^#^ *p* < 0.05, ^###^ *p* < 0.001 compared with the MK-801 treated group, respectively

MK-801, an NMDA receptor antagonist, co-administered with PF significantly delayed the onset time and decreased the total number of clonic seizure-like excitations induced by high dose morphine (250 nmol, i.t) in a dose-dependent manner. Moreover, PF administered with MK-801 not only significantly delayed onset but also decreased the total number of clonic seizure-like excitation (*p* < 0.001) (Fig. 5a and b).

### Effects of PF and antisense ODN of NMDA receptor subunits on high dose morphine-induced clonic seizure-like excitation behavior

**Fig. 6:**
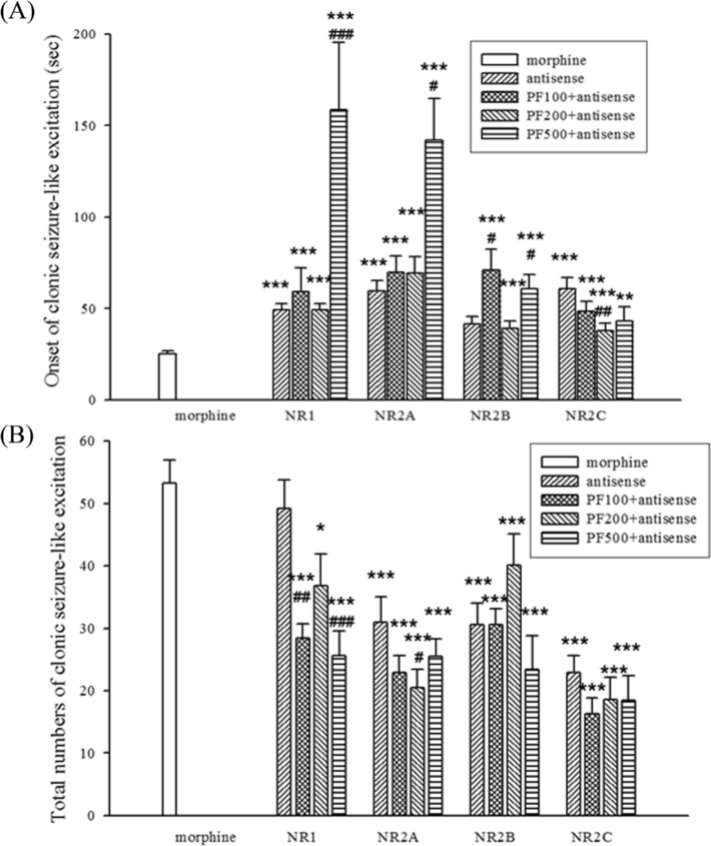
Effect of PF and antisense ODN of NMDA receptor subunits on high dose morphine-induced clonic seizure-like excitation in mice. Antisense ODN subunits: NR1, NR2A, NR2B, NR2C (15nM, i.c.v.) were administered 24 h before morphine (250 nmol, i.t.) administration. PF (100, 200, 500 nmol, i.c.v.) was administered 15 min before morphine administration. **a**: onset, **b**: total numbers of morphine-induced clonic seizure-like excitation during the first 60 min after morphine administration was recorded. Data were shown as mean ± S.E. (*n* = 12). ^*^ *p* < 0.05, ^***^ *p* < 0.001 compared with the morphine group. ^#^ *p* < 0.05, ^##^ *P* < 0.01, ^###^ *P* < 0.001 compared with itself antisense group

Administration of antisense ODN of NMDAR subunits: NR1, NR2A (2A), NR2B (2B), NR2C (2C) (15nM, i.c.v.) delayed the onset and decreased the total number of the high dose morphine-induced clonic seizure-like excitation compared with the morphine control group in mice (Fig. 6). PF (500 nmol) significantly delayed the onset of the high dose morphine-induced clonic seizure-like excitation when PF combined with ODN subunits, except NR2C (Fig. 6a). PF enhanced the inhibition of NR1 (at 100 and 500 nmol) and NR2A (at 200 pmol) on high dose morphine-induced clonic seizure-like excitation in mice (Fig. 6b).

### Effects of PF and antisense ODN subunits of NMDA receptor on NMDA-induced biting and scratching behavior

**Fig. 7:**
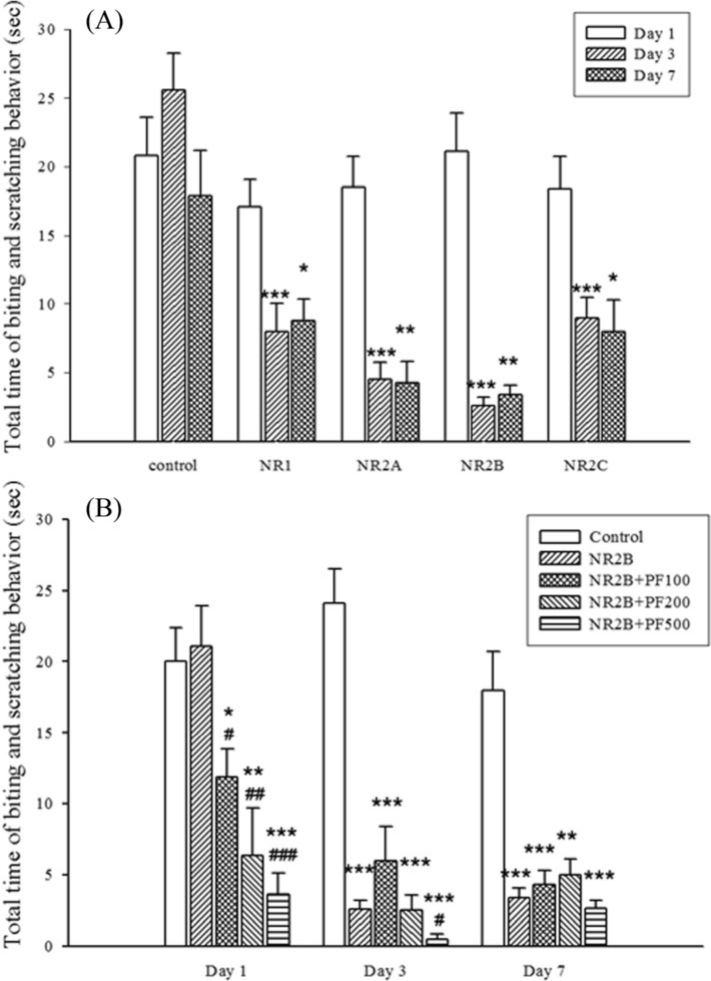
Effects of PF and antisense ODN subunits of NMDA receptor on the NMDA-induced biting and scratching behavior in mice. **a**: The time course effect of antisense ODNs on 1, 3, 7 days. Antisense ODNs: NR1, NR2A, NR2B, NR2C (15nM, i.c.v.) were administered 1, 3, 7 days before NMDA (122 pmol, i.t.). **b**: The effects of 2B and 2B combined with PF on biting and scratching behavior in NMDA-treated mice. PF 100, 200, 500 nmol, i.c.v.) were administered 15 min before of NMDA. Antisense ODNs (15 nM, i.c.v.) were administered 1, 3, 7 days before NMDA administration. The time spent on biting or scratching behavior during the first 2 min after NMDA administration was recorded. Data are shown as mean ± S.E. (*n* = 12). ^*^ *p* < 0.05, ^**^ *p* < 0.01, ^***^ *p* < 0.001 compared with the NMDA group; ^#^ *P* < 0.05, ^##^ *P* < 0.01, ^###^ *P* < 0.001 compared with the antisense 2B group, respectively

The inhibitory effect of PF (100, 200, 500 nmol) on NMDA-induced biting and scratching behavior was dose-dependent as shown in Fig. 7. Long term uses of antisense ODN subunits: NR1, NR2A, NR2B, NR2C (15nM, i.c.v.) significantly inhibited NMDA-induced biting and scratching behavior on days 3 and 7 (Fig. 7a). PF potentiated inhibition of NB2B on day 1 (at 100 nmol) and day 7 (at 500 nmol) (Fig. 7b).

### Effects of PF and antisense ODN NR2B subunit on NR2B-receptor abundance in mouse cortex

**Fig. 8:**
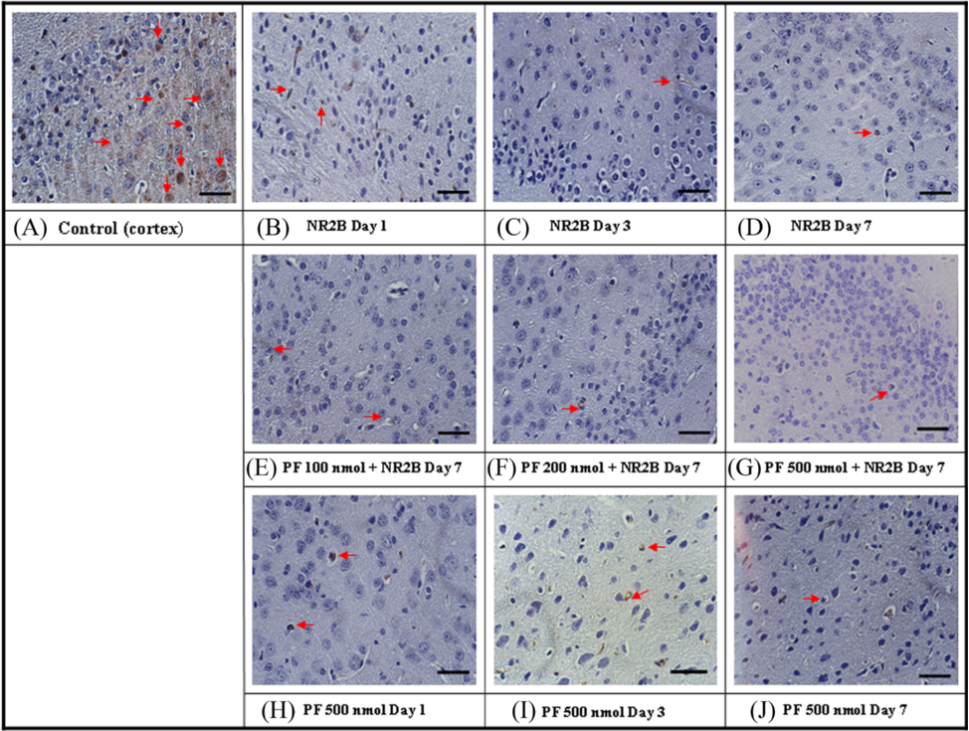
Effects of PF and antisense ODN of NMDA receptor subunit, NR2B on the NMDA receptor in mouse cortex by immunohistochemical assay. Antisense ODN subunit of NMDA receptor, NR2B (15 nM, i.c.v.) was administered once daily for 1–7 days. PF (100, 200, 500 nmol, i.c.v.) was administered 15 min before mice were sacrificed. The arrow was showing the NR2B positive cells. **a**: Normal control; **b**-**d**): treated with NR2B Day 1–7; **e**-**f**: PF 100–500 nmol combined treatment with NR2B in 7 days; **h**-**j**: treated with PF 500 nmol Day 1 ~ 7. Scare bar: 30 μm. (400×)

Mice treated with PF, and antisense ODN NR2B was used to determine the impact of PF on NR2B receptor abundance in mouse cortex. Immunohistochemical staining techniques detected protein receptors. Antisense ODN NR2B (15nM, i.c.v.) reduced NMDA receptor levels on days 1, 3, and 7. PF (500 nmol) reduced expression of the NR2B NMDA receptor (Fig. 8h-j). When the mouse was treated with antisense ODN NR2B and PF (at 100, 200, 500 nmol) showed a significant reduction of NR2B receptor expression on days 1, 3, and 7 in mouse cortex (Fig. 8b-g).

### Effects of PF and antisense ODN NR2B subunit on protein expression of NMDA receptor

**Fig. 9:**
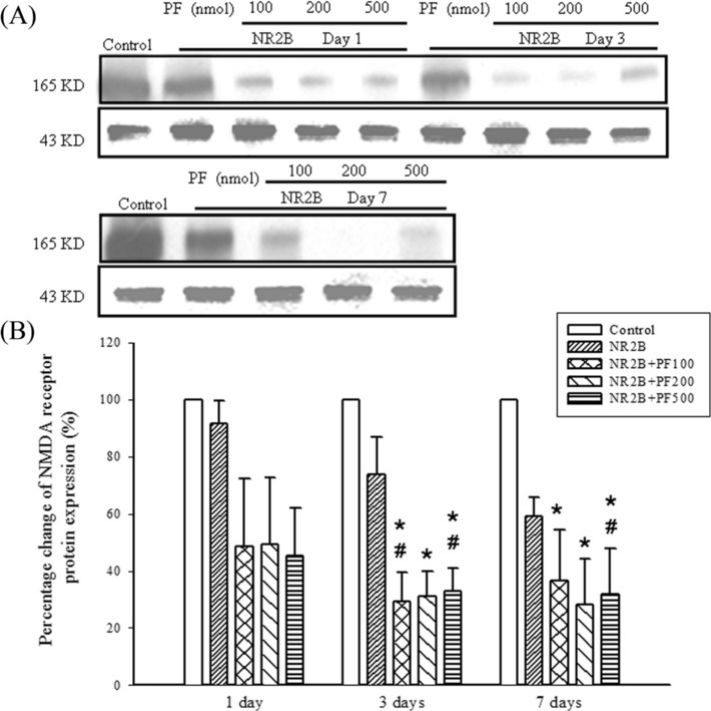
Effects of PF and antisense ODN NR2B on NMDA receptor protein expression in mouse brain. Antisense ODN, NR2B (15 nmol, i.c.v.) was administered 1, 3, 7 days. PF (500 nmol, i.c.v.) was administered 15 min prior to antisense ODN, NR2B. Protein level of NR2B was analyzed by Western blotting. β-actin was used as a control of protein loading. ^*^ *p* < 0.05 compared with the control group, ^#^ *p* < 0.05 compared with the antisense NR2B group itself on different days

Effects of PF and antisense ODN NR2B subunit in protein expression of the NMDA receptor were evaluated by using Western blotting. Co-administration of PF (200, 500 nmol) and antisense ODN NR2B significantly potentiated the reducing impacts of antisense ODN NR2B on NMDA NR2B subunit protein expression on day 3 and day 7 (Fig. 9, *p* < 0.05).

### Effects of PF docked in the active sites of NMDA receptor subtypes

**Fig. 10:**
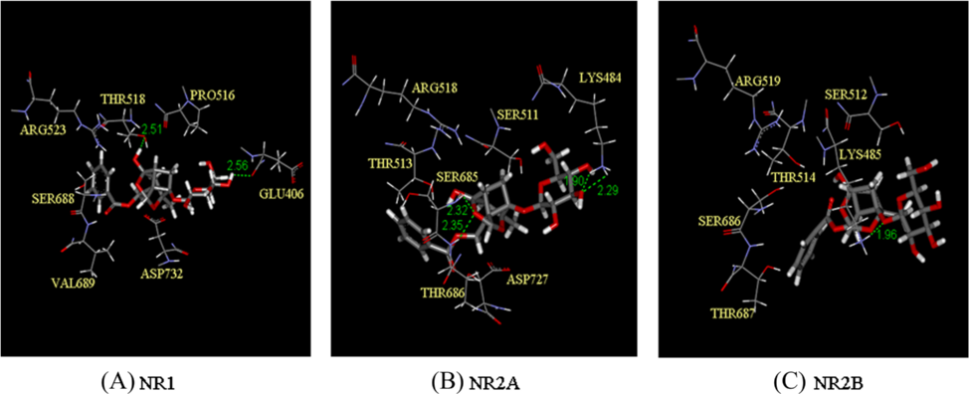
Effects of PF docked in the active sites of NMDA receptor subtypes. **a**: NR1 gaining −59.53 kcal/mol binding energy, which had one hydrogen bond with Glu406 (2.56 Å) and one with Thr518 (2.51 Å). **b**: NR2A gaining −128.49 kcal/mol binding energy, which had two hydrogen bonds with Lys484 (1.90 Å and 2.29 Å), one with Ser685 (2.32 Å) and one with Thr686 (2.35 Å). **c**: NR2B gaining −106.58 kcal/mol binding energy, which had one hydrogen bond with Lys485 (1.96 Å)

NR1 was gaining −59.53 kcal/mol binding energy, which had 1 hydrogen bond with Glu406 (2.56 Å) and one with Thr518 (2.51 Å). NR2A was gaining −128.49 kcal/mol binding energy, which had 2 hydrogen bonds with Lys484 (1.90 Å and 2.29 Å), one with Ser685 (2.32 Å) and one with Thr686 (2.35 Å). NR2B was gaining −106.58 kcal/mol binding energy, which had 1 hydrogen bond with Lys485 (1.96 Å) (Fig. 10).

## Discussion

Paeoniflorin, an active component from *Paeonia lactifloria*, is converted to paeonimetabolines by human intestinal bacteria [47]. Only very trace amount of paeoniflorin in rat hippocampus was detected by high-performance liquid chromatography after intravenous administration of *Paeoniae* Radix extract [48] Since the bioavailability for oral administration of paeoniflorin is low, the intracerebroventricular injection is applied in the present study to acquire high potency of paeoniflorin. Previously, it was reported that PF inhibited formalin-induced nociception in mice [32]. It has been suggested that formalin-induced nociception could be blocked by NMDA antagonists [13, 49]. In the present study, we determined if PF could inhibit formalin-induced licking and biting behavior by acting on the NMDA receptor.

PF produced significant inhibition of both neurogenic (early phase) and inflammatory (late phase) pain responses caused by formalin injection in mice. Co-administration of PF increased the antinociceptive effect of MK-801 on formalin-induced nociception. The enhancement of PF on the inhibitory effects of MK-801 on formalin-induced licking behavior is significant both in early phase and in late phase.

However, PF did not affect 100 nmol *trans*-(±) - ACPD-induced nociceptive behavior (data not shown). Since *tran*s-(±)-ACPD is an mGluRs agonist, effects of PF effects may be mediated primarily by acting on iGluRs but not mGluRs.

To provide more direct evidence concerning the interaction of PF with iGluRs, we determined if PF could diminish EAA agonist-induced nociceptive behavior in mice. PF attenuated biting and scratching behavior induced by glutamate, NMDA, and AMPA in a dose-dependent manner. Co-administration of PF augmented effects of MK-801 on reducing nociceptive behavior induced by the 3 EAA agonists. MK-801 is a NMDA receptor antagonist in the glutamate category involved with the central nervous system [50]. Co-administration of PF and MK-801 revealed a synergistic inhibitory effect on formalin-induced licking behavior. The effect observed from the combination of PF and MK-801 might be due to their effect on NMDA receptor with different binding sites.

Our results indicate that the antinociception effects of PF are due in part to targeting the glutamatergic system, especially via interaction with iGluRs. Such conclusion is supported by evidence showing that PF produced significant attenuation of the biting and scratching behavior induced by NMDA, AMPA, and glutamate. Besides, the spinal NMDA receptors may be primarily involved in eliciting the licking and biting behavior that followed intrathecal injection of high-dose morphine.

PF not only delayed the onset but also decreased the total number of clonic seizure-like excitation induced by morphine, the antinociception caused by PF was due to an interaction with iGluRs, more specifically via an interaction with the NMDA receptor.

Impaired glutaminergic neurotransmission has been implicated in several neurological diseases such as acute stroke, trauma, epilepsy, schizophrenia, depression, chronic pain and opioid dependence [51]. The excitatory effect of glutamate is thought to be mediated in part through NMDA receptors [3, 4, 10]. Substances which block NMDA receptors could have potential clinical use in pain management. However, antagonists that completely block NMDA receptors cause numerous side effects, such as ataxia, motor incoordination, memory impairment and psychotomimetic effects. Therefore, developing new substances that can inhibit excitation, and maintain the normal physiological function of NMDA receptors would be ideal candidates for the treatment of NMDA-induced diseases. Such agents might be predicted to be devoid of CNS side effects at doses producing potent antinociception at peripheral NMDA receptors [52]. The antinociception effects of PF on amino acid agonists and high-dose morphine-induced nociceptive behavior are mediated by targeting iGluRs, especially modulation of the NMDA 2B receptor. The NMDA receptor family consists of NR1, NR2 (A, B, C, and D), and NR 3 (A and B) subunits, which determine the characteristics of native NMDA receptors. Among NMDA receptor subunits, the NR2B subunit for pain is particularly noteworthy. A drug that has NR2B selective antagonistic properties, may be effectively used for the treatment of chronic pain [24]. In this study, it revealed that NR2B was significantly inhibited by PF and antisense ODN for NR2B. PF significantly increased the suppressive effects of the antisense oligodeoxynucleotide (ODN) subunits, NR1, NR2A, NR2B on EAA and high dose morphine-induced nociceptive behavior. PF targeting NR2A and NR2B subunits was confirmed in *in silico* experiments. Docking energy data revealed that paeoniflorin had stronger binding activity in NR2A and NR2B than NR2C of NMDA receptors. Notably, the result of docking procedure was corresponding to the experiment of an animal model.

Remarkably, no side effects such as ataxia, motor incoordination, memory impairment and psychomimetic effects were found in mice during the period of antisense ODNs of NMDAR subunits co-administered with PF. This observation reveals the superiority of PF.

## Conclusions

**Fig. 11:**
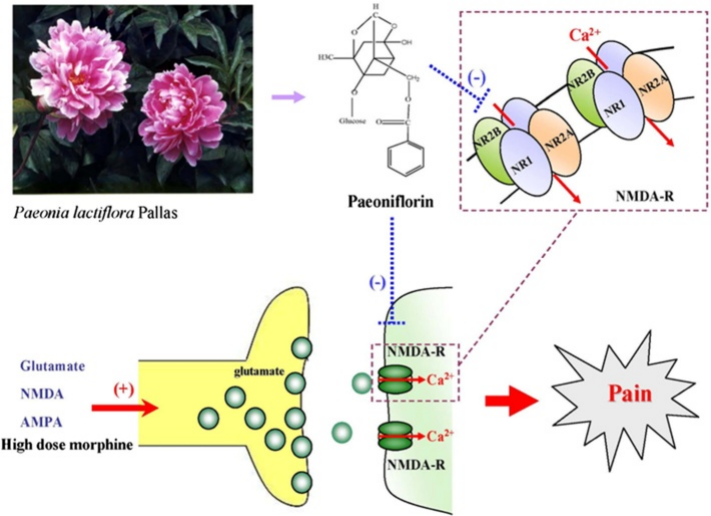
Paeoniflorin inhibits excitatory amino acid agonist- and high-dose morphine- induced nociceptive behavior in mice via modulation of NMDA receptor

In conclusion, this present study indicates that PF counteracts nociceptive behavior via modulation of NMDA receptor for the first time (Fig. 11). Although the precise site of action of PF remains to be determined, targeting of the NMDA 2B receptor by PF may play a significant role in suppression of nociception.

## Abbreviations

ACSF, artificial CSF; AMPA, α-amino-3-hydroxy-5-methylisoxazole-4-propionic acid; EAA, excitatory amino acid; GluRs, glutamate receptor; Glutamate, L-glutamic acid hydrochloride; i.c.v., intracerebroventrical injection; i.t., intrathecal injection; IACUC, institutional animal care and use committee; iGluRs, iontropic glutamate receptors; mGluRs, metabotropic glutamate receptors; MK-801, (5R,10S)-(+)-5-methyl- 10,11-dihydro-5H-dibenzo [*a*,*d*] cyclohepten-5,10-imine hydrogen maleate; NBQX, 2,3-Dioxo-6-nitro-1,2,3,4-tetrahydrobenzo [*f*] quinoxaline-7-sulfonamide disodium; NMDA, *N*-methyl-D-asparate; NMDAR, NMDA receptor; ODN, oligodeoxynucleotide; PF, paeoniflorin; PVDF, polyvinylidene fluoride
